# The importance of RT-qPCR primer design for the detection of siRNA-mediated mRNA silencing

**DOI:** 10.1186/1756-0500-4-148

**Published:** 2011-05-25

**Authors:** Mike Herbert, Natacha Coppieters, Annette Lasham, Helen Cao, Glen Reid

**Affiliations:** 1 Genesis Research & Development Corporation, Ltd, PO Box 50, Auckland 1140, New Zealand; 2 Department of Pharmacology, Faculty of Medical and Health Sciences, University of Auckland, Auckland, New Zealand; 3 Department of Structural Biology, School of Biological Sciences, University of Auckland, Auckland, New Zealand; 4 Department of Pharmacology, University of Auckland, Auckland, New Zealand; 5 Department of Molecular Medicine and Pathology, Faculty of Medical and Health Sciences, University of Auckland, Auckland, New Zealand; 6 CSL Limited, 45 Poplar Rd, Parkville VIC 3052, Australia; 7 Asbestos Diseases Research Institute (ADRI), Bernie Banton Centre, University of Sydney, Hospital Road, Concord NSW 2139, Australia

## Abstract

**Background:**

The use of RNAi to analyse gene function *in vitro *is now widely applied in biological research. However, several difficulties are associated with its use *in vivo*, mainly relating to inefficient delivery and non-specific effects of short RNA duplexes in animal models. The latter can lead to false positive results when real-time RT-qPCR alone is used to measure target mRNA knockdown.

**Findings:**

We observed that detection of an apparent siRNA-mediated knockdown *in vivo *was dependent on the primers used for real-time RT-qPCR measurement of the target mRNA. Two siRNAs specific for *RRM1 *with equivalent activity *in vitro *were administered to A549 xenografts via intratumoural injection. In each case, apparent knockdown of *RRM1 *mRNA was observed only when the primer pair used in RT-qPCR flanked the siRNA cleavage site. This false-positive result was found to result from co-purified siRNA interfering with both reverse transcription and qPCR.

**Conclusions:**

Our data suggest that using primers flanking the siRNA-mediated cleavage site in RT-qPCR-based measurements of mRNA knockdown *in vivo *can lead to false positive results. This is particularly relevant where high concentrations of siRNA are introduced, particularly via intratumoural injection, as the siRNA may be co-purified with the RNA and interfere with downstream enzymatic steps. Based on these results, using primers flanking the siRNA target site should be avoided when measuring knockdown of target mRNA by real-time RT-qPCR.

## Background

The use of RNAi to inhibit gene expression has revolutionised medical research and has great therapeutic potential. However, inefficient siRNA delivery and off-target effects hamper translation from *in vitro *experiments to *in vivo *research and clinic application. Many approaches to improve delivery are under investigation, such as the use of localised delivery by direct injection and topical application, and intravenous administration for systemic delivery [1-3]. Despite the growing use of RNAi *in vivo*, very few studies include data to confirm that the observed effects of the siRNA are due to an RNAi-mediated mRNA cleavage mechanism rather than non-specific events.

The importance of confirming that mRNA reduction following siRNA administration has occurred via RNAi-mediated events is highlighted by recent studies reporting the contribution of the innate immune system to apparent *in vivo *knockdown of target mRNAs. The double-stranded nature of siRNA imparts the ability to trigger an innate immune response via the activation of Toll-like receptors (TLR 3, 7 and 8) and binding to proteins such as retinoic acid inducible gene 1 (RIG-1) [4,5]. These interactions may cause a down-regulation of gene expression that can be falsely attributed to a sequence-specific RNAi-meditated event. Together this suggests that many of the reports of *in vivo *efficacy of siRNAs can be explained by a general down-regulation of transcription that is stimulated by the double-stranded RNA structure of siRNA without involving RNAi, especially in the absence of corroborating evidence [6,7].

More recently Holmes *et al. *found that the 3'fragment produced following siRNA-mediated cleavage of certain target mRNAs can persist and that this can compromise RT-qPCR-mediated detection of knockdown [8], similar to the findings of others [9,10] suggesting that incomplete degradation of mRNA cleavage fragments can result in inaccurate determination of knockdown by RT-qPCR. They suggest the use of primers flanking the cleavage site as a means to avoid this problem. Here we show that this approach can lead to artefactual results when siRNAs are used in certain *in vivo *settings, as siRNAs co-purified with total RNA can interfere with downstream analysis, in some cases leading to false positive results.

## Materials and methods

### Cell Culture

The A549 (human non-small cell lung cancer) and Hepa 1-6 (mouse hepatoma) cell lines used in this study were obtained from ATCC and were grown in RPMI medium supplemented with 10% heat-inactivated fetal bovine serum (FBS) (both from Invitrogen Corporation, Carlsbad, CA), at 37°C in humidified air with 5% CO_2_.

### siRNAs and transfection

The siRNAs and Lipofectamine RNAiMax were from Invitrogen. The siRNA sequences are listed in Table 1. Transfection was carried out as described previously [11], using Lipofectamine RNAiMax (at a concentration of 0.8 μL per mL) and cells at a final density of 5 × 10^3 ^per cm^2^. After overnight incubation, transfection medium was replaced with RPMI containing 10% FBS, and cells were analyzed at indicated time points.

**Table 1:** Sequences of siRNAs, RNA oligos and RT-qPCR primers used in the study

*siRNAs*		
*Name*	*Passenger strand*	*Guide strand*

RRM1-2	CCCAGUUACUGAAUAAGCAGAUCUU	AAGAUCUGCUUAUUCAGUAACUGGGCU
RRM1-3^§^	GCAAACUCACUAGUAUGCACUUCUA	UAGAAGUGCAUACUAGUGAGUUUGCCU
ApoB-1^§^	GUCAUCACACUGAAUACCAAU	AUUGGUAUUCAGUGUGAUGAmC*mA*C
ApoB1 mm control	GUGAUCAGACUCAAUACGAAU	AUUCGUAUUGAGUCUGAUCAmC*mA*C
81-control	AAGAUCUGCUUAUUCAGUAACUGGG	CCCAGUUACUGAAUAAGCAGAUCUU

***Single-strand RNA oligos***		

*Name*	*Sequence*	

RRM1-3-sense	GCAAACUCACUAGUAUGCACUUCUA	
RRM1-3-antisense	UAGAAGUGCAUACUAGUGAGUUUGCCU	

***qPCR Primers***		

*Target*	*Forward*	*Reverse*

RRM1(A)^†^	TGGATCAAGGTGGGAACAAG	CGACGAGAAGGAAAGGACAC
RRM1(B)^†^	GGTACAAGGTCTGGCAGATGCT	TTCCAGTGTCGACCGAAGGT
RRM1(C)^†^	CATCCACATTGCTGAGCCTAAC	GGGTCAGAAGTTTGGGACGAA
ApoB1 site	AGCCATGGGCAACTTTACCT	AAAGGAAATGGGCAACGATA
ApoB external	GGCACTGTGGGTCTGGAT	TTCTTCTCTGGAGGGGACTG
HMBS	AGCCTGTTTACCAAGGAGCT	GAGTGAACAACCAGGTCCAC
LMNA	TGAGGCCAAGAAGCAACTTCA	CTCATGACGGCGCTTGGT
Polr2a	TTACTCCCCTGCATGGTCTC	TGGGAGACATAGCACCACCT

**Figure 1:**
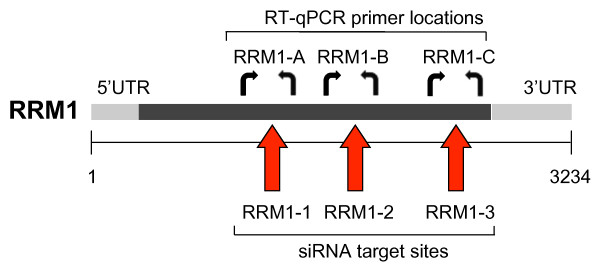
**Schematic representation of *RRM1 *mRNA, showing siRNA target sites and primer pairs**. Red arrows indicate siRNA target sites and PCR primers are represented by curved arrows.

* Represents phosphorothioate linkages between nucleotides in the siRNA backbone. ^† ^Targets RRM1-A, B and C represent the cleavage sites for the various *RRM1*-specific siRNAs, as shown schematically in Figure 1A. ^§^RRM1-3 and ApoB1 are complementary in sequence to both human and mouse target genes. Accession numbers: *RRM1*, NM_001033.3; *LMNA*, NM_005572.3; *HMBS*, NM_000190.3; *ApoB*, NM_009693.2; *Polr2a*, NM_009089.2. Amplicon sizes: RRM1(A): 114 bp; RRM1(B): 127 bp; RRM1(C): 79 bp; LMNA: 139 bp; HMBS: 65 bp; ApoB1 site 107 bp; ApoB external: 86 bp; Polr2a: 95 bp

### RT-qPCR

RNA was isolated using the PureLink 96 RNA Purification system (Invitrogen), with an additional Trizol (Invitrogen) extraction for *in vivo *samples, quantified via spectrophotometry using a Nanodrop (Thermo Scientific), and first-strand cDNA synthesised using Superscript III polymerase (Invitrogen), as described [12]. For real-time qPCR, the cDNA was diluted 1:4 in 10 mM Tris pH 7.0, with reactions carried out on a LightCycler 480 (Roche) using SybrGreen I Master mix (Roche) and gene-specific primers at 180 nM (Table 1) in 384 white multi-well plates [12]. Primer sets specific to different regions were used to measure *RRM1 *mRNA, as depicted in Figure 1. Melting curve analysis was performed to confirm that a single product was being amplified, and the products were run out on agarose gels to confirm their predicted amplicon size (data not shown). For competition experiments, the indicated concentrations of siRNAs or RNA oligonucleotides were added to the RNA or cDNA templates prior to reverse transcription or PCR, respectively. Levels of *RRM1 *mRNA were normalised to the reference genes *LMNA *or *HMBS*, with *ApoB *normalised to *Polr2a *for mouse samples, and relative change in mRNA levels following treatment with specific or control siRNA was calculated from triplicate technical replicates of each using the 2^-ΔΔCt ^method [13]. RNA, tumour and cell samples were all stored at -80°C, whilst cDNA was stored at -20°C. Positive controls for RT-qPCR using cDNA prepared from an A549 *in vitro *culture showed % CV of < 1.1 for all human primer sets between assays, whilst intra-assay variation was < 1% for human primers, and < 2% for mouse primers.

### MBRACE

5'-RLM-RACE was performed using the GeneRacer kit (Invitrogen) with the manufacturer's instructions modified as previously described [12]. The first round 5'RACE reaction product (1 μl) was used as a template for the MBRACE reaction [12] using the FastStart TaqMan^® ^Probe Master (Roche) and primers and probes at the following concentrations: 180 nM MB-R, 3.6 μM MB-F and 250 nM molecular beacon probe (all specific for the target gene cleavage site). Reactions were run on a LightCycler 480 as described [12].

### Tumour models

A549 or Hepa 1-6 cells grown *in vitro *were detached from flasks with trypsin, and the enzymatic reaction was stopped by the addition of fresh culture medium containing FBS. After two washes with PBS, the cells were resuspended in PBS at a final density of 8 × 10^7 ^cells/ml (A549 cells) or 5 × 10^6 ^cells/ml (Hepa1-6 cells). Using a 26-gauge needle, groups of five CD1 nude mice were injected subcutaneously on the flank with 100 μl of the cell suspension. When tumours reached 50-100 mm^3 ^in size, they were twice injected (24 h apart) with 25 μg siRNA in 50 μl saline; tumours were excised as described (12). The effect of intratumoural injection on *RRM1 *or *ApoB *mRNA levels was assessed by real-time RT-qPCR 24 h after the second injection.

## Results and Discussion

### *RRM1 *reduction measured by RT-qPCR following intratumoural siRNA administration is primer-pair specific

In a previous screen we identified *RRM1 *as a potential target for siRNA-based cancer therapy [11]. Three different siRNAs (RRM1-1, -2 and -3) induced significant knockdown of *RRM1 *mRNA and protein in A549 cells *in vitro*, leading to growth inhibition and the induction of apoptosis [11]. Furthermore, transfection of A549 cells with RRM1-2 prior to implantation into nude mice markedly inhibited tumour growth [11]. Having observed growth inhibitory effects both *in vitro *and *in vivo *following *RRM1 *knockdown in tumour cells, we then assessed the ability of siRNAs RRM1-2 and RRM1-3 to silence *RRM1 *mRNA via intratumoural injection of siRNAs into pre-existing subcutaneous xenografts. These siRNAs were shown to be equally potent with IC_50 _values *in vitro *of approximately 20 pM [11,14]. Tumours were twice injected (24 h apart) with 25 μg of RRM1-2 or RRM1-3 siRNA in 50 μl normal saline and excised 24 h after the second injection. RNA was isolated and *RRM1 *expression analysed by RT-qPCR using two different primer pairs (RRM1-B and RRM1-C in Figure 1).

**Figure 2:**
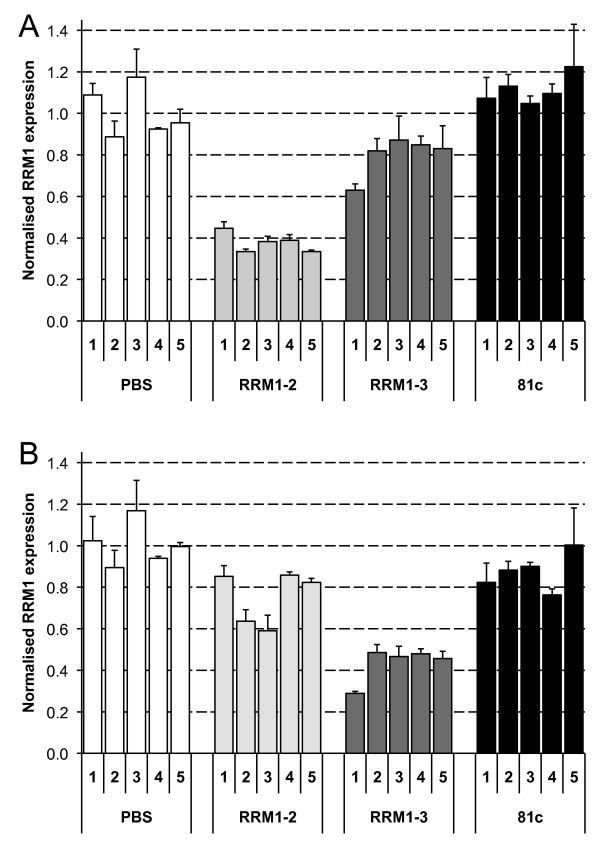
**Apparent knockdown of RRM1 mRNA measured by RT-qPCR following intratumoural injection of siRNA**. A549 xenograft tumours were injected with PBS, RRM1-2, RRM1-3 or non-targeting siRNA (81c) (n = 5). *RRM1 *mRNA was measured by real-time RT-qPCR. PCR was carried out with primers flanking the RRM1-2 (RRM1-B, shown in (A)) or RRM1-3 site (RRM1-C, shown in (B)).

As seen in Figure 2, measurement of an apparent change in mRNA levels was dependent on location of the primer pair in relation to the siRNA target site (shown schematically in Figure 1). Primers flanking the RRM1-2 siRNA target site (RRM1-B) showed an apparent reduction of *RRM1 *mRNA of up to 60% in tumours injected with RRM1-2 siRNA, whereas little knockdown was observed in tumours injected with RRM1-3 siRNA (Figure 2A). Conversely, the use of a primer pair flanking the RRM1-3 site (RRM1-C) in real-time RT-qPCR showed a much greater apparent reduction in tumour *RRM1 *mRNA levels in tumours injected with RRM1-3 siRNA (Figure 2B). However, the use of primer pair RRM1-A, generating an amplicon upstream of both RRM1-2 and RRM1-3 target sites resulted in measurements of RRM1 mRNA levels that did not differ from control siRNA-injected tumours (data not shown).

**Figure 3:**
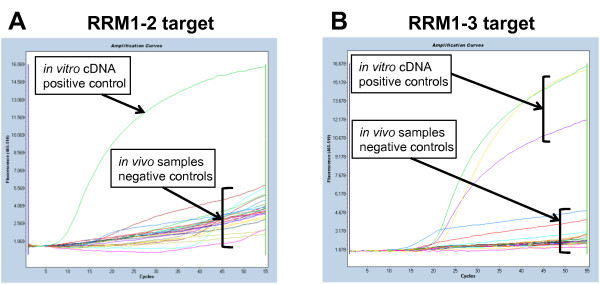
**Detection of siRNA-mediated cleavage of *RRM1 *mRNA following *in vitro *transfection or intratumoural injection with RRM1 siRNAs**. A549 xenograft tumours were injected as described in Figure 2 with RRM1-2 siRNA (A) or RRM1-3 siRNA (B). *In vitro *positive control: cDNA from A549 cells transfected with 1 nM RRM1-2 or RRM1-3 siRNA; samples were tested in triplicate, one single amplification curve is shown per sample for clarity.

**Figure 4:**
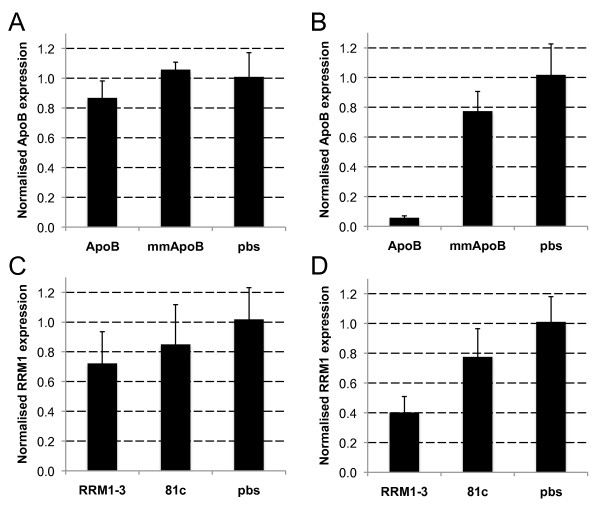
**Apparent knockdown of mRNA in mouse tumours is primer pair-specific**. Hepa 1-6 tumours (n = 5) were injected with the indicated siRNAs and *ApoB *(A, B) or *Rrm1 *(C, D) mRNA levels measured by real-time RT-qPCR using primers flanking the siRNA target site (B, D) or outside this region (A,C). mmApoB is an inactive mismatched siRNA control; 81C is a control with no known mRNA target, pbs - PBS-injected tumours.

These results suggested that the knockdown was non-specific, and this was further confirmed using a Molecular Beacon RACE assay, MBRACE, [12] specific for the cleavage point of either RRM1-2 or RRM1-3 siRNAs. Cleavage of *RRM1 *mRNA was detected with cDNA prepared from RNA isolated from A549 cells transfected with either RRM1-2 and RRM1-3 siRNA, but despite the significant knockdown detected by RT-qPCR in the *in vivo *study (Figure 2) there was no detection of specific products of siRNA-mediated knockdown in injected tumours (Figure 3). Similar results were observed when Hepa1-6 tumours were injected with *ApoB*- or *Rrm1*-specific siRNA. The ApoB1 siRNA used was previously shown to silence *ApoB *effectively *in vitro*, as well as *in vivo *following hydrodynamic tail-vein injection [12]. After intratumoural injection, however, an apparent reduction in the *ApoB *or *Rrm1 *mRNA was observed only when the primers flanked the target site (Figure 4).

### The presence of siRNA compromises downstream reactions

The apparent knockdown of *RRM1/Rrm1 *in tumours following intratumoural injection of siRNA was found to relate to the primer pair used in real-time RT-qPCR, in contrast to the similar levels of knockdown measured *in vitro *after transfection with three different siRNAs, irrespective of the primer pair used in RT-qPCR. This suggested that the residual siRNA was interfering with subsequent steps in the analysis, perhaps explained by the concentration of siRNA in the RNA isolated in each system. The method of RNA isolation used in this study involves binding the RNA to a size selection column, which should exclude small RNAs less than 90 bp. However, miRNAs have been isolated using this procedure [15] suggesting that the columns only reduce, but do not exclude, small RNAs.

In the *in vitro *transfection, an siRNA concentration of 10 nM is the equivalent to ~80 ng per well in a 24-well plate. If one assumes cells take up half of the siRNA used in the transfection (and minimal degradation occurs during the 24 h transfection period), the siRNA component of the RNA isolated from the cells (around 10 μg) is less than 1% of the total (80 ng siRNA in ~10 μg of cell-derived total RNA). In contrast, 25 μg siRNA injected twice into a tumour with a volume of 50 mm^3 ^and yielding 50 μg total RNA is likely to be a much greater proportion of the isolated RNA and has the potential to interfere with downstream applications. Further adding to the potential for co-purification is the use of Stealth-modified siRNA duplexes, which have chemical modifications imparting resistance to nucleases and stability in serum.

**Figure 5:**
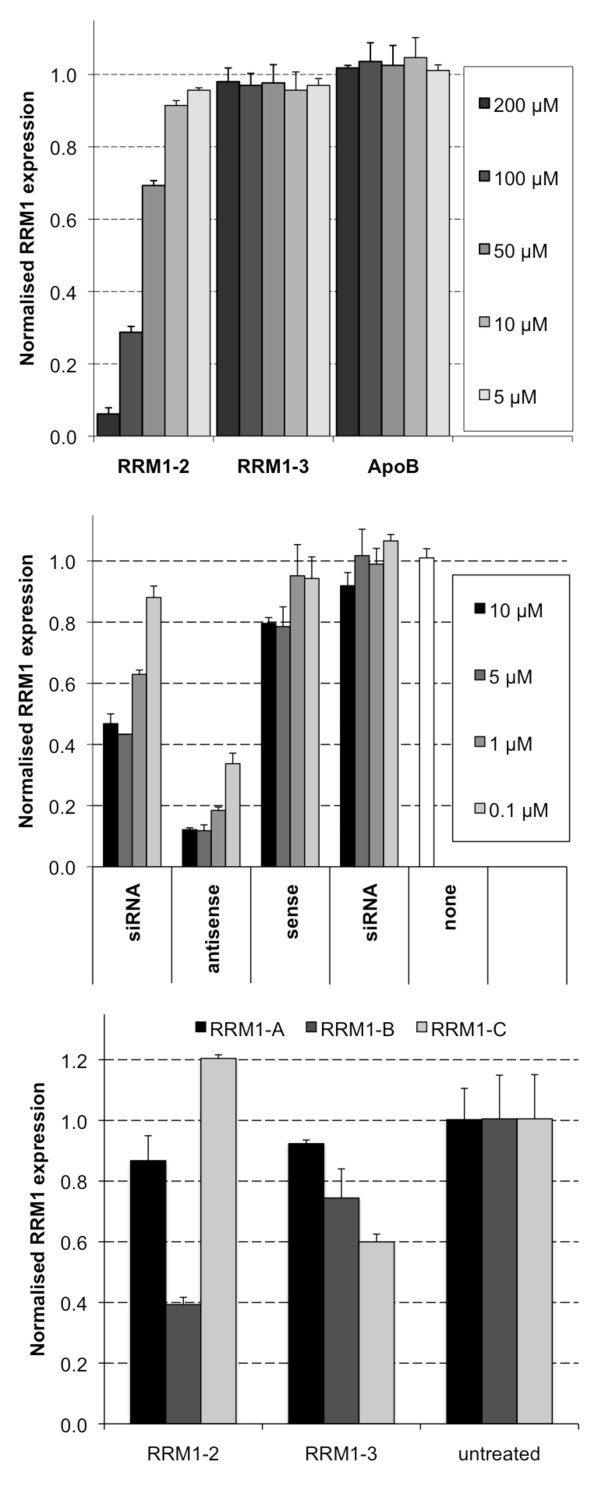
**Effect of siRNA on mRNA measurements when added to various steps of real-time RT-qPCR**. (A) The siRNAs RRM1-2, RRM1-3 and ApoB1 were added at the indicated concentration (μM) to the cDNA template prior to real-time qPCR using primers flanking the RRM1-2 site (RRM1-B). (B) The siRNA, anstisense or sense strands of RRM1-3, or RRM1-2 siRNA were added at the indicated concentration (μM) to the RNA isolated from the tumour of a PBS-treated mouse prior to reverse transcription. (C) RRM1-2 or RRM1-3 siRNA (25 μg) or water was added to tumours before RNA isolation and *RRM1 *mRNA was quantified using primer sets RRM1-A or RRM1-B.

To explore this possibility, we investigated the effect of adding siRNA to the real-time qPCR step (Figure 5). We used cDNA reverse-transcribed from RNA isolated from the tumour of a PBS-treated mouse as template for real-time qPCR, and measured the effects of adding increasing amounts of various siRNAs. As shown in Figure 5A, PCR with the addition of RRM1-2 siRNA at high (50 to 200 μM) concentrations led to inhibition of RT-qPCR, but only when the primer pair flanking the target site (RRM1-B) was used. In contrast, there was no effect on the *RRM1 *mRNA levels measured in the presence of either RRM1-3 or *ApoB*-specific siRNAs.

We also assessed whether siRNA was able to interfere with the reverse transcription step of real-time RT-qPCR. We again used RNA isolated from the tumour of a PBS-treated mouse as template, but here we added increasing concentrations of siRNA or single-stranded RNA to the RNA template in the reverse transcription reaction. RRM1-3 or control siRNA, as well as the single-stranded sense or antisense strands of the RRM1-3 siRNA were added at the concentrations indicated. As seen in Figure 5B, introducing either the RRM1-3 siRNA duplex or antisense strand led to reduced detection of *RRM1 *message when RRM1-3 flanking primers (RRM1-C) were used. In contrast, introducing RRM1-2 siRNA or RRM1-3 sense strand had no effect on measurements of *RRM1 *levels. When the primer pair flanking the RRM1-1 site (RRM1-A) was used, no interference was observed (data not shown). Lastly, we added RRM1-2 or RRM1-3 siRNA (25 μg) to tumours prior to RNA extraction, and evaluated mRNA measurements with different primer pairs (Figure 5C). Only when a flanking primer pair was used with template containing the corresponding siRNA was an apparent knockdown detected; no effect was seen with the RRM1-A primer pair, which is located upstream of both RRM1-2 and RRM1-3 sites.

## Conclusions

Following intratumoural injection of *RRM1*-specific siRNAs, the apparent reduction of *RRM1 *transcript levels was found to be a function of the primer pair used. Subsequent *in vitro *investigations suggested that this most likely resulted from interference with reverse transcription, and to a lesser extent real-time qPCR, caused by siRNA co-purified in the RNA isolation. These data suggest that primers flanking the siRNA target site should be avoided in studies of siRNA *in vivo*, especially when large amounts of siRNA are used.

## Competing interests

The authors declare that they have no competing interests.

## Authors' contributions

MH performed molecular studies and analysed data, NC designed competition experiments, AL designed experiments and analysed data, HC carried out tumour studies, GR conceived of the study and drafted the manuscript. All authors read and approved the final manuscript.
